# Cardiovascular drugs and COVID‐19 clinical outcomes: a systematic review and meta‐analysis of randomized controlled trials

**DOI:** 10.1111/bcp.15331

**Published:** 2022-04-25

**Authors:** Innocent G. Asiimwe, Sudeep P. Pushpakom, Richard M. Turner, Ruwanthi Kolamunnage‐Dona, Andrea L. Jorgensen, Munir Pirmohamed

**Affiliations:** ^1^ The Wolfson Centre for Personalised Medicine, MRC Centre for Drug Safety Science, Department of Pharmacology and Therapeutics, Institute of Systems, Molecular and Integrative Biology University of Liverpool Liverpool UK; ^2^ Department of Health Data Science, Institute of Population Health Sciences, University of Liverpool, United Kingdom Institute of Population Health Sciences University of Liverpool Liverpool UK

**Keywords:** cardiovascular drugs, COVID‐19, living systematic review, meta‐analysis, RCTs

## Abstract

**Aims:** To update our previously reported systematic review and meta‐analysis of observational studies on cardiovascular drug exposure and COVID‐19 clinical outcomes by focusing on newly published randomized controlled trials (RCTs).

**Methods:** More than 500 databases were searched between 1 November 2020 and 2 October 2021 to identify RCTs that were published after our baseline review. One reviewer extracted data with other reviewers verifying the extracted data for accuracy and completeness.

**Results:** After screening 22 414 records, we included 24 and 21 RCTs in the qualitative and quantitative syntheses, respectively. The most investigated drug classes were angiotensin‐converting enzyme inhibitors (ACEIs)/angiotensin receptor blocker (ARBs) and anticoagulants, investigated by 10 and 11 studies respectively. In meta‐analyses, ACEI/ARBs did not affect hospitalization length (mean difference −0.42, 95% confidence interval [CI] −1.83; 0.98 d, *n* = 1183), COVID‐19 severity (risk ratio/RR 0.90, 95% CI 0.71; 1.15, *n* = 1661) or mortality (risk ratio [RR] 0.92, 95% CI 0.58; 1.47, *n* = 1646). Therapeutic anticoagulation also had no effect (hospitalization length mean difference −0.29, 95% CI −1.13 to 0.56 d, *n* = 1449; severity RR 0.86, 95% CI 0.70; 1.04, *n* = 2696; and, mortality RR 0.93, 95% CI 0.77; 1.13, *n* = 5689). Other investigated drug classes were antiplatelets (aspirin, 2 trials), antithrombotics (sulodexide, 1 trial), calcium channel blockers (amlodipine, 1 trial) and lipid‐modifying drugs (atorvastatin, 1 trial).

**Conclusion:** Moderate‐ to high‐certainty RCT evidence suggests that cardiovascular drugs such as ACEIs/ARBs are not associated with poor COVID‐19 outcomes, and should therefore not be discontinued. These cardiovascular drugs should also not be initiated to treat or prevent COVID‐19 unless they are needed for an underlying currently approved therapeutic indication.

## 1 INTRODUCTION

Cardiovascular diseases, mainly ischaemic heart disease, stroke and heart failure, were the leading causes of global mortality in 2017, accounting for approximately 17.8 million deaths.^1^ By contrast, the coronavirus disease 2019 (COVID‐19) pandemic^2^, ^3^ has killed >5.5 million people (out of approximately 335 million infected) as of 19 January 2022.^4^ Due to the possible bidirectional interaction between COVID‐19 and cardiovascular disease,^5^, ^6^ we conducted a baseline systematic review and meta‐analysis^7^ to evaluate the available evidence on the association between cardiovascular drug exposure and COVID‐19 clinical outcomes. Additionally, and because COVID‐19 related evidence is rapidly evolving, we planned for periodic updating for up to 2 years to incorporate any novel evidence.

In the baseline review (search date 1 November 2020), we included 429 and 390 studies in the qualitative and quantitative syntheses, respectively, with the majority of these being observational studies (only 2 randomized control trials [RCTs]).^7^ In the adjusted estimates, angiotensin‐converting enzyme inhibitors (ACEIs)/angiotensin receptor blockers (ARBs), the most commonly reported drug classes, were not associated with COVID‐19 infectivity (odds ratio [OR] 0.92, 95% confidence interval [CI] 0.71 to 1.19), hospitalization (OR 0.93, 95% CI 0.70 to 1.24), severity (OR 1.05, 95% CI 0.81 to 1.38) or mortality (OR 0.84, 95% CI 0.70 to 1.00). However, and even though adjustment may account for some confounders present in observational studies, it does not account for nonmeasured confounders and pooling of adjusted estimates may be problematic.^8^, ^9^ For more reliable evidence, therefore, a decision was made a priori to focus on RCTs, the current gold standard of evidence,^10^ in this update.

In this update, we report RCT evidence for 7 cardiovascular drug classes/subclasses, namely: angiotensin‐converting enzyme inhibitors (ACEIs), angiotensin receptor blockers (ARBs), anticoagulants, antiplatelets, antithrombotics, calcium channel blockers (CCBs) and lipid‐modifying drugs (LMDs). Although evidence is still emerging, all these drug classes/subclasses have been implicated in modulating the outcomes of COVID‐19. Specifically, it has been suggested that ACEIs and ARBs, through their respective modes of action, increase the expression of angiotensin‐converting enzyme 2 (ACE2).^11^, ^12^ ACE2 converts angiotensin (Ang) II into the vasodilator and antitrophic heptapeptide, Ang‐(1–7), which exerts protective effects on the lung and cardiac vasculature making it potentially beneficial in COVID‐19 patients.^5^, ^12^, ^13^ Although both ACEIs and ARBs increase ACE2 expression, Ang II levels are decreased with ACEIs,^12^ which means there will be less substrate for ACE2 to convert into Ang‐(1–7). Consequently, ARBs may be better than ACEIs at attenuating inflammation and acute lung injury in COVID‐19 patients.^14^ In contrast to these protective effects, SARS‐CoV‐2, the virus that causes COVID‐19, uses ACE2 to enter target cells,^15^ which led to the hypothesis that ACEIs/ARBs could modulate COVID‐19 disease outcomes. COVID‐19 patients have increased haemostatic and thrombotic risks—these have been postulated to originate from the development of a cytokine storm after SARS‐CoV‐2 infection, followed by hyperinflammation, endothelial disruption, platelet activation and coagulopathy among other mechanisms.^16^, ^17^ By helping avert haemostatic and thrombotic complications, anticoagulants, antiplatelets and other antithrombotic agents could therefore positively impact COVID‐19 clinical outcomes such as hospitalization, severity and mortality. By contrast, CCBs may interfere with SARS‐CoV‐2 replication by reducing intracellular calcium levels, and the resulting anti‐inflammatory (reduction of COVID‐19 related inflammation), anticoagulatory (reduction of microvascular coagulation) and vasodilatory (improvement of local vasoconstriction) effects, may decrease COVID‐19 severity and associated mortality.^18^ Lastly, LMDs have also been reported to possess antiviral, immunomodulatory, anti‐inflammatory and antithrombotic properties,^19^, ^20^ which may all contribute to better COVID‐19 clinical outcomes.

## 2 METHODS

We followed a predefined protocol (PROSPERO: CRD42020191283^21^) as previously reported.^7^ This manuscript follows the Preferred Reporting Items for Systematic Reviews and Meta‐Analyses (PRISMA) guidelines^22^ (Table S1).

### 2.1 Identification of studies

As previously detailed,^7^, ^21^, ^23^ we searched >500 databases (including MEDLINE and Scopus) through the University of Liverpool's DISCOVER platform, preprint servers, and COVID‐19 specific databases/registries, but this time focused on RCTs published/posted between 1 November 2020 and 2 October 2021. To the DISCOVER search strategy (previously only included medical subject headings and text words related to “cardiovascular drugs” and “COVID‐19”), we added additional terms (random* OR RCT* OR [clinical AND trial*]) to further limit the search results. The DISCOVER search results were uploaded in EndNote (version X9)^24^ and studies were de‐duplicated based on title, author, year of publication and reference type information. We also hand‐searched the lists of references from the identified studies and previous systematic reviews, although no additional eligible articles were identified. Any additional studies published after 2 October 2021 and referred to us (e.g. by experts) before data analysis were also included.

### 2.2 Selection criteria

We included RCTs that investigated the association between cardiovascular drug exposure (key drug classes were derived from Chapter 2 [“Cardiovascular system”] of the British National Formulary^25^ as previously outlined^7^) and the COVID‐19 clinical outcomes listed below. We excluded non‐English studies but did not exclude any studies based on where and when they were published.

### 2.3 Outcomes

For those at risk of COVID‐19, the clinical outcome that we considered was COVID‐19 infectivity (defined as a positive COVID‐19 diagnosis) while for COVID‐19 patients, we considered hospitalization, hospitalization length, severity and all‐cause mortality.^7^

### 2.4 Study selection and data extraction

I.G.A. screened titles and abstracts of all retrieved records, and thereafter retrieved full texts of the potentially eligible studies. I.G.A. used an adapted data extraction form^7^ to extract information related to study design, characteristics of included patients/investigated drugs, COVID‐19 outcomes and study risk of bias. Either S.P. or R.M.T. verified the accuracy and completeness of the extracted data, with any disagreements being resolved by consensus.

### 2.5 Assessment of study quality

I.G.A. used the revised Cochrane risk‐of‐bias tool for randomized trials^26^ to assess the quality of each included study estimate, with S.P./R.M.T. verifying accuracy, and disagreements being resolved by consensus.

### 2.6 Data synthesis

The R meta package^27^ (R version 3.6.1) was used to pool count data (preferred to summary estimates such as the odds ratios, as it is more accurate^8^) in cases where 2 or more studies reported on the same exposure–outcome combination. The pooling was done using random‐effects meta‐analysis with the inverse‐variance method for effect size and the DerSimonian–Laird estimator for variance.^27^ For the dichotomous outcomes (infectivity, hospitalization, severity and mortality), we generated risk ratios (RR; with 95% confidence intervals [CIs]) as odds ratios are not easily interpretable^28^ and case control studies were not included (in which RRs, unlike odds ratios, are not applicable). Mean differences (with 95% confidence intervals) were generated for hospitalization length, the only continuous outcome. Where median values and ranges/interquartile ranges of hospitalization length were reported, we used them to estimate the means and standard deviations.^29^, ^30^ Means and standard deviations could also be combined using formulae available in the Cochrane Handbook.^30^ Lastly, we prepared Forest plots for each exposure–outcome combination and narratively reported/tabulated the studies that singly reported on an exposure–outcome combination, as part of the qualitative synthesis.

### 2.7 Heterogeneity measures

As previously reported,^7^ the magnitude of the inconsistency in the study results was assessed by visually examining forest plots and considering the *I* ^2^ statistic (arbitrarily defined heterogeneity extent categories were: *I* ^2^ < 30%, low; *I* ^2^ = 30–70%, moderate; and *I* ^2^ > 70%, high). Estimates with high heterogeneity were deemed to be inconsistent and would result in a downgrading of the strength of evidence.^31^

### 2.8 Publication bias

We did not assess publication bias since there were fewer than 10 studies for each of the reported exposure–outcome combinations.

### 2.9 Subgroup and sensitivity analyses

We conducted subgroup analyses based on drug subclasses, study quality (only studies with low risk of bias included) and the hypertension comorbidity, which was guided by our earlier findings.^23^ Where a study reported different estimates for the same outcome domain (for example due to different follow‐up periods), we conducted sensitivity analyses to determine the impact of the inclusion of 1 estimate, instead of the other(s).

### 2.10 Confidence in cumulative evidence

We used the GRADE (Grading of Recommendations, Assessment, Development and Evaluations)^31^ criteria to rate the strength of the body of evidence.

### 2.11 Nomenclature of Targets and Ligands

Key protein targets and ligands in this article are hyperlinked to corresponding entries in http://www.guidetopharmacology.org, and are permanently archived in the Concise Guide to PHARMACOLOGY 2019/20.^32^

## 3 RESULTS

### 3.1 Study selection and characteristics

**FIGURE 1:**
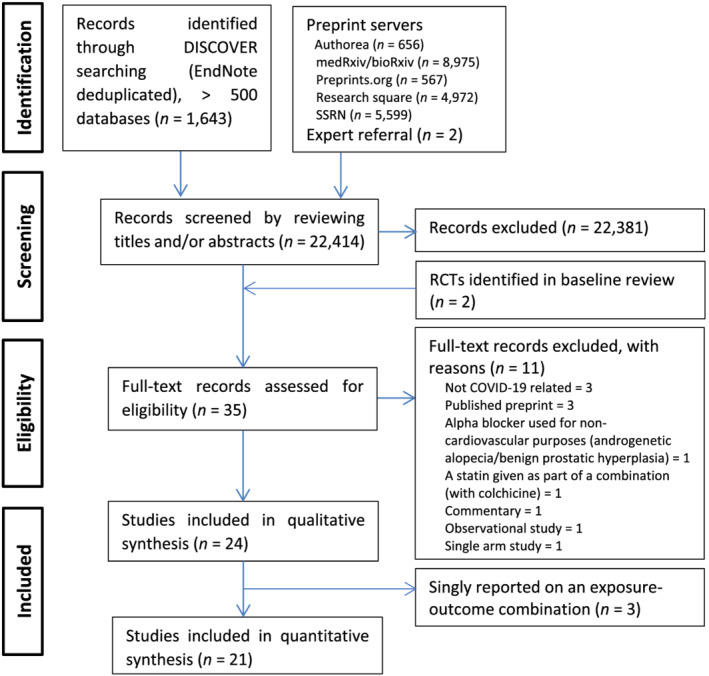
PRISMA Flow Chart of Included Studies. Abbreviations: SSRN = Social Science Research Network, RCT = randomized controlled trial

After screening 22 414 titles and/or abstracts, 35 full‐text records were assessed for eligibility, of which 24 and 21 were included in the qualitative and quantitative syntheses, respectively (Figure 1). Table 1 shows the characteristics of the included studies. Of the 24 studies, most (*n* = 23, 95.8%) are now published in peer‐reviewed journals with only 1 (4.2%) still being a preprint. These studies used patients recruited from 21 countries, including Argentina, Australia, Austria, Bolivia, Brazil, Canada, Colombia, Germany, Indonesia, Iran, Ireland, Mexico, Nepal, Netherlands, Peru, Saudi Arabia, Spain, Sweden, United Arab Emirates, UK and USA. Two of the studies^44^, ^45^ reported data that was obtained from the same patients, although the follow‐up periods (30 *vs*. 90 d) were different. When 1 of these studies is not counted, from a total of 23 studies, most were prospectively registered (*n* = 22, 95.7%), were of multicentre (*n* = 19, 82.6%) and open‐label (*n* = 16, 69.6%) designs, and recruited a median sample size of 205 (range 20–14 892) patients. Based on the estimate‐specific revised Cochrane risk‐of‐bias tool (assesses the risk of bias of a single trial result/assessment at outcome‐level),^26^ all study estimates were rated as having a low risk of bias except for estimates from 6 studies^33^, ^34^, ^38^, ^41^, ^42^, ^51^ (Table S2).

**TABLE 1:** Characteristics of included studies

No.	First Author (Trial acronym)	Peer‐reviewed (Published /posted date)	Country	Design	Registry number	Recruited from	Recruitment period	Eligibility ^a^	Previous drug use allowed	Sample size (I *vs*. C)	Race, % (I *vs*. C)	Age (y),mean ± SD or median (IQR) (I *vs*. C)	Male, % (I *vs*. C)	HTN/DM, % (I *vs*. C)	Baseline severity (I *vs*. C)	Intervention (I *vs*. C)	Follow‐up time	Outcomes	Main results (OR/HR/RR, d or number of events ^b^, I *vs*. C, with *P*‐value, if reported)
***ACEIs***																			
1	Amat‐Santos, IJ^33^ (RASTAVI)	Yes (26‐May‐20)	Spain	Multicentre open‐label parallel 1:1 RCT (retrospective analysis of RCT data)	NCT03201185	14 Spanish centres	1 Jan–1 Apr 2020	Adult aortic stenosis patients successfully treated TAVR	No (in previous 3 mo)	102 (50 *vs*. 52)	NI	82 ± 6 (both arms)	57 (both arms)	54/21 (both arms)	Not yet infected	Ramipril (initial dose 2.5 mg/d, titrated up to 10 mg/d) *vs*. conventional treatment	Median time on treatment was 6 (IQR 2.9–11.4) mo.	Infectivity	HR 1.150 (95%CI 0.351–3.768)
***ARBs***																			
2	Nouri‐Vaskeh, M^34^	Yes (13‐Mar‐21)	Iran	Single‐centre open‐label parallel 1:1 RCT	IRCT20180802040678N4	Imam Reza Hospital, Tabriz	2 Apr–30 Jun 2020	Hospitalised adult COVID‐19 patients with primary HTN	No	80 (41 *vs*. 39)	All Iranian (Asian)	67 ± 15 *vs*. 60 ± 17	54 (both arms	100/27 *vs*. 100/21	NI (all inpatient)	Losartan 25 mg twice daily *vs*. amlodipine (a CCB), 5 mg/d for 2 wk)	30 d	Hospitalization length	4.57 ± 2.59 *vs*. 7.30 ± 8.70 d, *P* = .085
																		Severity	8 *vs*. 9 intubations
																		Mortality	2 *vs*. 5 deaths, *P* = .241
3	Guyatt, GH^35^	Yes (11‐May‐21)	USA	Multicentre open‐label parallel 1:1 RCT	NCT04340557	Sharp Memorial Hospital, Sharp Grossmont Hospital and Sharp Chula Vista Hospital in Southern California	30 Mar–4 Jul 2020	Hospitalized adult COVID‐19 patients with mild hypoxaemia (within 72 h of SARS‐CoV‐2 nucleic acid testing confirmation)	No	31 (16 *vs*. 15)	75% Hispanic, 6% White, 6% Black, 13% Unknown *vs*. 87% Hispanic, 13% White	59 *vs*. 55 (mean) or 53 in both (median)	63 *vs*. 60	44/19 *vs*. 33/33	All with mild to moderate hypoxia (SpO_2_ ≤ 96% on ≥1 L/min oxygen by nasal cannula) but not on MV	Losartan 12.5 mg twice daily for up to 10 d or until hospital discharge, with the option to titrate upward dependent on blood pressure tolerability) plus SoC *vs*. SoC alone	Until discharge or until an endpoint was met in the hospital	Hospitalization length	Mean 9 (range 3–30) *vs*. 10 (range 3–34) d
																		Severity	1 *vs*. 2 ICU admissions
																		Mortality	1 *vs*. 1 in‐hospital deaths
4	Geriak, M^36^	Yes (17‐Jun‐21)	USA	Multicentre double‐blind placebo‐controlled parallel 1:1 RCT	NCT04311177	MHealth Fairview, Hennepin Healthcare and Mayo Clinic in Minnesota	Apr–Nov 2020	Symptomatic adult COVID‐19 outpatients (enrolled within 7 d of symptom onset)	No	117 (58 *vs*. 59)	78% White, 7% Black, 2% Asian, 9% Hispanic, 5% other/unknown *vs*. 66% White, 7% Black, 9% Asian, 9% Hispanic, 10% other/unknown	38 (29–51) *vs*. 37 (27–46)	43 *vs*. 58	10/7 *vs*. 5/5	All symptomatic outpatients	Losartan 25 mg twice daily (once daily for those with eGFR 30–60 mL/min/1.73 m2) *vs*. placebo for 10 d	28 d	Hospitalization	3 (5.2%) *vs*. 1 (1.7%) hospitalizations, absolute difference of 3.5%, 95% CI −4.8–13.2%, *P* = .320
																		Severity	1 *vs*. 1 ICU admissions
																		Mortality	0 *vs*. 0 deaths
5	Puskarich, MA^37^	No (28‐Aug‐2021)	USA	Multicentre double‐blind placebo‐controlled parallel 1:1 RCT	NCT04312009	13 US hospitals	Apr 2020–Feb 2021	Symptomatic adult hospitalized COVID‐19 patients with a respiratory sequential organ failure assessment score of at least 1.	No	205 (101 *vs*. 104)	35% White, 37% Black, 7% Asian, 19% Hispanic, 3% other/unknown *vs*. 45% White, 29% Black, 2% Asian, 17% Hispanic, 7% other/unknown	54 ± 16 *vs*. 56 ± 15	59 *vs*. 61	37/21 *vs*. 44/25	20% ED, 58% floor, 5% step‐down or intermediate, 17% ICU *vs*. 19% ED, 61% floor, 12% step‐down or intermediate, 9% ICU	Losartan 50 mg twice daily (once daily for those with eGFR 30–60 mL/min/1.73 m2) *vs*. placebo for 10 d	28 d	Severity	89 *vs*. 94 required oxygen
																			21/100 *vs*. 17/103 intubations
																		Mortality	11 *vs*. 9 deaths
																	90 d	Mortality	11 *vs*. 11 deaths
6	Duarte, M^38^	Yes (18‐Jun‐21)	Argentina	Multicentre open‐label parallel 1:1 RCT with adaptive design aspects	NCT04355936	University of Buenos Aires main hospital and a community hospital	14 May–30 Oct 2020	Hospitalized adult COVID‐19 Patients (4 or fewer d since symptom onset)	No	158 (78 *vs*. 80)	NI	64 ± 17 *vs*. 67 ± 17	63 *vs*. 44	45/21 *vs*. 44/18	71 *vs*. 66% required supplementary oxygen at admission (ICU patients not enrolled)	Telmisartan 80 mg twice daily for 14 d plus SoC *vs*. SoC alone	30 d	Hospitalization length	Median of 9 *vs*. 15 d (log‐rank *P* < .0001)
																		Severity	9 *vs*. 24 ICUs/MVs/deaths (*P* = .0058)
										141 (70 *vs*. 71)								Mortality	3 *vs*. 16 deaths (*P* = .0023)
***ACEI/ARBs***																			
7	Cohen, JB^39^ (REPLACE COVID)	Yes (07‐Jan‐21)	USA, Peru, Argentina, Bolivia, Canada, Mexico, Sweden	Multicentre open‐label parallel 1:1 RCT	NCT04338009	20 large referral hospitals in 7 countries worldwide	31 Mar–20 Aug 2020	Hospitalized adult COVID‐19 patients who were receiving ACEI/ARB therapy as an outpatient before admission	Yes	152 (75 *vs*. 77)	53 Hispanic/Latino, 16 White, 13 Black, 17 other *vs*. 55 Hispanic/Latino, 14 White, 16 Black, 16 other	62 ± 12 *vs*. 62 ± 12	56 *vs*. 55	100/56 *vs*. 100/48	88% mild/ moderate, 12% severe *vs*. 87% mild/moderate, 13% severe—WHO severity definition	ARB/ACEI continuation *vs*. discontinuation (at previously prescribed doses)	28 d	Hospitalization length	Median 6 (IQR 3–11) d *vs*. 5 (3–10) d
																		Severity	16 *vs*. 14 ICU admissions/invasive MVs
																		All‐cause death	11 *vs*. 10 deaths
8	Lopes, RD^40^ (BRACE CORONA)	Yes (19‐Jan‐21)	Brazil	Multicentre registry‐based open‐label parallel 1:1 RCT	NCT04364893	29 hospitals	9 Apr–26 Jun 2020	Hospitalized adult COVID‐19 patients (mild to moderate severity)	Yes	659 (325 *vs*. 334)	NI	56 (46–66) *vs*. 55 (46–63)	60 *vs*. 59	100/31 *vs*. 100/33	All mild/moderate ^c^	ARB/ACEI continuation *vs*. discontinuation	30 d	Hospitalization length	6.7 ± 6.3 *vs*. 7.8 ± 7.4 d
																		Severity (COVID‐19 progression)	105 *vs*. 128 with worsened clinical severity
																		All‐cause mortality	9 *vs*. 9 deaths
9	Bauer, A^41^ (ACEI‐COVID)	Yes (11‐Jun‐21)	Austria and Germany	Multicentre open‐label parallel 1:1, RCT	NCT04353596	35 centres (19 university clinics and 16 large referral hospitals)	20 Apr 2020–20 Jan 2021	Adults with recent symptomatic SARS‐CoV‐2 infection (≤ 5 d) and were chronically treated with ACEI/ARBs before admission	Yes (treated for ≥ 1 mo)	204 (100 *vs*. 104)	100% White	75 (69–80) *vs*. 74 (63–80)	64 *vs*. 63	99/37 *vs*. 96/29	78 *vs*. 89% needed oxygen/respiratory therapy	ARB/ACEI continuation *vs*. discontinuation	30 d	Hospitalization length (d)	11.00 (6.75–19.00) *vs*. 10.00 (5.75–15.25) d, *P* = .27
																		Severity	26 *vs*. 21 ICU admissions/MVs/deaths, *P* = .41
																		Mortality	12 *vs*. 8 deaths, *P* = .42
10	Najmeddin, F^42^	Yes (15‐Jul‐21)	Iran	Multicentre parallel triple‐blind RCT	IRCT20151113025025N3	3 academic hospitals affiliated to Tehran University of Medical Sciences	Apr–Sep 2020	Hospitalized adult COVID‐19 patients with HTN treated with ACEIs/ARBs	Yes	57 (28 *vs*. 29)	NI	65 ± 10 (for 31) *vs*. 68 ± 10 (for 33)	55 (for 31) *vs*. 39 (for 33)	100/48 (for 31) *vs*. 100/52 (for 33)	All moderate to severe (based on national definitions)	ARB/ACEI continuation *vs*. discontinuation (ARBs/ACEIs replaced by amlodipine ± carvedilol according to the dose equivalents)	14 d	Hospitalization length	5.3 ± 3.9 *vs*. 8.0 ± 15.9 d (4 [2–8] *vs*. 4 [2–5] d also reported)
																		Severity	4 *vs*. 3 invasive MVs
																			4 *vs*. 7 ICU admissions
																			5 *vs*. 7 ICU admissions/MVs
																			19 *vs*. 20 with WHO COVID‐19 ordinal endpoint ≥ 6
																		Mortality	5 *vs*. 4 deaths
***Anticoagulants***																			
11	Lemos, ACB^43^ (HESACOVID)	Yes (21‐Sep‐20)	Brazil	Single centre open‐label phase II RCT	REBEC RBR‐949z6v	Ribeirão Preto School of Medicine, São Paulo University	Apr–Jul 2020	Adult COVID‐19 patients with ARDS and requiring MV	No	20 (10 *vs*. 10)	NI	55 ± 10 *vs*. 58 ± 16	90 *vs*. 70	40/40 *vs*. 30/30	All required MV	Therapeutic enoxaparin *vs*. standard anticoagulant thromboprophylaxis as per protocol.	28 d	Hospitalization length	31 (22–35) *vs*. 30 (23–38) d, *P* = .838
																		Mortality	2 *vs*. 5 in‐hospital deaths, *P* = .160 (1 *vs*. 3 all‐cause deaths, *P* = .264)
12	Sadeghipour, P^44^ (INSPIRATION)	Yes (18‐Mar‐21)	Iran	Multicentre open‐label 2 × 2 factorial design RCT	NCT04486508	10 academic centres in Tehran and Tabriz	29 July–19 Nov 2020	ICU adult COVID‐19 patients (admitted to ICU within 7 d of initial hospitalization—with expected survival of at least 24 hours)	No	562 (276 *vs*. 286)	All likely Asian	62 (51–71) *vs*. 61 (47–71)	59 *vs*. 57	48/30 *vs*. 41/26	All in ICU	Intermediate‐dose (enoxa parin, 1 mg/kg daily) *vs*. standard‐dose (enoxaparin, 40 mg daily) prophylactic anticoagulation for 30 d, with protocol‐guided modifications.	30 d	Hospitalization/ICU length	5 (2–10) *vs*. 6 (3–11) d, *P* = .14)
																		All‐cause mortality	119 *vs*. 117 deaths, *P* = .50)
13	Bikdeli, B^45^ (INSPIRATION)	Yes (17‐Apr‐21)															90 d	All‐cause mortality	127 *vs*. 123 deaths; HR 1.24 95% CI 0.97–1.60, *P* = .11
14	Lopes, RD^46^ (ACTION)	Yes (04‐Jun‐21)	Brazil	Multicentre pragmatic open‐label parallel 1:1 RCT	NCT04394377	31 sites	24 Jun 2020–26 Feb 2021	Hospitalized adult COVID‐19 patients with elevated D‐dimer concentration (with COVID‐19 symptoms for up to 14 d before randomisation)	Yes (<48 h use and could be stopped at study entry).	614 (310 *vs*. 304)	NI	57 ± 14 *vs*. 57 ± 15	62 *vs*. 58	49/27 *vs*. 50/22	10% mild, 83% moderate, 8% severe *vs*. 13% mild, 82% moderate, 5% severe ^d^	Therapeutic (rivaroxaban 15/20 mg with or without enoxaparin or unfractionated heparin, depending on clinical stability) *vs*. prophylactic (standard in‐hospital enoxaparin or unfractionated heparin) anticoagulation.	30 d	Hospitalization length	8.1 ± 7.2 *vs*. 7.8 ± 7.5 d, *P* = 0.96
																		All‐cause mortality	35 *vs*. 23 deaths; RR 1.49 (0.90–2.46), *P* = 0.13
15	Perepu, US^47^	Yes (08‐Jul‐21)	USA	Multicentre open‐label parallel 1:1, RCT	NCT04360824	University of Iowa, Gunderson Health System and Louisiana State University	26 Apr 2020–6 Jan 2021	Hospitalized adult severe COVID‐19 patients (admitted to an ICU and/or having laboratory evidence of coagulopathy)	No (excluded if they had an indication for therapeutic dose anticoagulation)	173 (87 *vs*. 86)	78% White, 10% Hispanic, 8% Black, 2% Asian, 1% Other *vs*. 73% White, 17% Hispanic, 3% Black, 2% Asian, 3% Other	65 (range 24–86) *vs*. 64 (30–85)	54 *vs*. 58	59/34 *vs*. 62/40	All were either admitted to an ICU and/or had a modified ISTH Overt DIC score ≥3	Intermediate weight adjusted enoxaparin (1 mg/kg daily or 0.5 mg/kg twice daily if BMI ≥ 30 kg/m^2^) *vs*. standard prophylactic enoxaparin (40 mg daily or 30/40 mg twice daily if BMI ≥ 30 kg/m^2^)	30 d	All‐cause mortality	13 *vs*. 18 deaths; OR 0.66; 95% CI 0.30–1.45; *P* = .31
16	Sholzberg, M^48^ (RAPID COVID COAG)	Yes (14‐Oct‐21), first identified as a preprint	Brazil, Canada, Ireland, Saudi Arabia, United Arab Emirates, USA	Multicentre pragmatic open‐label parallel 1:1 RCT	NCT04362085; NCT04444700	28 sites in 6 countries	29 May 2020–12 Apr 2021	Hospitalized adult COVID‐19 patients with elevated D‐dimer levels (within the first 5 d of admission)	Yes (if on an intermediate dose of heparin that can be changed)	465 (228 *vs*. 237)	73% White, 12% Asian, 8% Black, 6% Hispanic or Latino, <1% other *vs*. 69% White, 16% Asian, 10% Black, 4% Hispanic or Latino, <1% other	60 ± 14 *vs*. 60 ± 16	54 *vs*. 60	47/36 *vs*. 49/33	Moderate (requiring ward‐level care with D‐dimer levels above the ULN in the presence of an oxygen saturation ≤93% on room air, or ≥2 times the ULN irrespective of oxygen saturation)	Therapeutic *vs*. prophylactic heparin as per protocol, administered until hospital discharge, d 28, study withdrawal or death.	28 d	Severity	37 *vs*. 52 ICU admissions/MVs/deaths, OR, 0.69; 95% CI, 0.43–1.10, *P* = .12
																		Mortality	4 *vs*. 18 deaths, OR 0.22; 95% CI, 0.07–0.65, *P* = .006
17	Goligher, EC^49^ (REMAP‐CAP, ACTIV‐4a and ATTACC)	Yes (4‐Aug‐21)	UK, USA, Canada, Brazil, Ireland, Netherlands, Australia, Nepal, Saudi Arabia, Mexico	Multiplatform open‐label adaptive parallel RCT	NCT02735707; NCT04505774; NCT04359277; NCT04372589	393 sites in 10 countries	21 Apr–19 Dec 2020	Hospitalized adult severe/ICU/critically ill COVID‐19 patients	No (excluded if they had a separate indication for therapeutic dose anticoagulation)	1098 (534 *vs*. 564)	74% White, 16% Asian, 6% Black, 4% Other (for 536) *vs*. 74% White, 16% Asian, 5% Black, 6% Other (for 567)	60 ± 13 *vs*. 62 ± 13	72 *vs*. 68	NI/32 *vs*. NI/34	All severe (required ICU‐level respiratory or cardiovascular organ support)	Therapeutic anticoagulation with heparin *vs*. pharmacological thromboprophylaxis as per local usual care for up to 14 d or hospital discharge/recovery.	28 d	Mortality	199 *vs*. 200 deaths
18	Lawler, PR^50^ (REMAP‐CAP, ACTIV‐4a and ATTACC)	Yes (4‐Aug‐21)	USA, Canada, UK, Brazil, Mexico, Nepal, Australia, Netherlands, Spain			121 sites in 9 countries	21 Apr 2020–22 Jan 2021	Hospitalized adult patients with moderate COVID‐19 (not critically ill)		2231 (1181 *vs*. 1050)	63% White, 4% Asian, 22% Black, 14% Other (for 1181) *vs*. 67% White, 5% Asian, 19% Black, 12% Other (for 1050)	59 ± 14 (for 1181) *vs*. 59 ± 14 (for 1050)	60 (for 1181) *vs*. 57 (for 1050)	53 (for 1023)/30 (for 1181) *vs*. 50 (for 892)/30 (for 1049)	All moderate (did not need ICU‐level care)		28 d	Severity	129 *vs*. 127 invasive MVs/deaths, adjusted OR = 0.82 (0.63–1.07).
										2221 (1175 *vs*. 1046)								Severity	243 *vs*. 257 received organ support or died, adjusted OR = 1.30 (1.05–1.61).
										2226 (1180 *vs*. 1046)								All‐cause mortality	86 *vs*. 86 deaths
19	Ananworanich, J^51^	Yes (15‐Sep‐21)	USA	Multicentre double‐blind placebo‐controlled parallel 1:1 RCT	NCT04504032	13 outpatient clinics in 7 states and at 1 virtual site that enrolled participants from 40 states	16 Aug 2020–3 Feb 2021	Adult symptomatic mild COVID‐19 patients at high‐risk for progression to severe COVID‐19 based on age, obesity, or a comorbidity.	No	444 (222 *vs*. 222)	89% White, 7% Black, 0% Asian, 4% other/unknown *vs*. 89% White, 7% Black, 1% Asian, 4% other/unknown	49 (range 20 −83) *vs*. 49 (18 −75)	43 *vs*. 37	48/26 *vs*. 56/30	1% asympto matic, 93% mild, 6% moderate/severe *vs*. < 1% asymptomatic, 98% mild, 2% moderate/severe (based on Gates MRI Ordinal Scale)	Rivaroxaban 10 mg *vs*. placebo (multivitamin supplement) once daily for 21 d	28 d	Hospitalization	3 *vs*. 7 hospitalizations (2/192 *vs*. 5/199 in those treated with at least 1 dose and with mild COVID‐19 at d 1)
																		Severity	46 *vs*. 44 progressed to moderate/severe disease (18/192 *vs*. 23/199 in those treated with at least 1 dose and with mild COVID‐19 at d 1)
																		Mortality	0/219 *vs*. 0/230 deaths
20	Spyropoulos, AC^52^ (HEP‐COVID)	Yes (07‐Oct‐21)^e^	USA	Multicentre open‐label 1:1 active control RCT	NCT04401293	12 academic centres	8 May 2020–14 May 2021	Hospitalized adult COVID‐19 patients who required supplemental oxygen and either D‐dimer levels >4 times the upper limit of normal or sepsis‐induced coagulopathy score of ≥4.	No (those with a need for full dose anticoagulation or dual antiplatelet therapy were excluded)	253 (129 *vs*. 124)	9% Asian, 26% Black, 43% White, 23% multiracial/unknown *vs*. 11% Asian, 30% Black, 37% White, 22% multiracial/unknown	66 ± 14 *vs*. 68 ± 14	53 *vs*. 55	63/40 *vs*. 57/35	35 *vs*. 31% in ICU	Therapeutic enoxaparin (0.5–1 mg/kg twice daily based on creatinine clearance) *vs*. institutional standard prophylactic or intermediate‐dose heparins throughout hospitalization.	30 d	Hospitalization length	12.2 ± 9.3 *vs*. 11.6 ± 8.2 d
																		Mortality	25 *vs*. 31 deaths, RR, 0.78; 95% CI, 0.49–1.23; P = .28.
***Antiplatelets***																			
21	Horby, PW^53^ (RECOVERY)	Yes (17‐Nov‐21), first identified as a preprint	UK, Indonesia, Nepal	Multicentre platform open‐label factorial RCT	ISRCTN 50189673; NCT04381936	177 hospitals (UK), 2 hospitals (Indonesia) and 2 hospitals (Nepal)	1 Nov 2020–21 Mar 2021	Hospitalized adult COVID‐19 patients	No (those currently receiving aspirin or another antiplatelet were excluded)	14 892 (7351 *vs*. 7541)	74% White, 16% Black/ Asian/minority ethnic, 10% unknown *vs*. 75% White, 16% Black/Asian/minority ethnic, 9% unknown (for 7351 *vs*. 7541)	59 ± 14 *vs*. 59 ± 14 (for 7351 *vs*. 7541)	62 *vs*. 61 (for 7351 *vs*. 7541)	NI/22 *vs*. NI/22 (for 7351 *vs*. 7541)	All hospitalized at baseline; 67% no respiratory support or simple oxygen, 28% noninvasive ventilation, 5% invasive MV *vs*. 67% no respiratory support or simple oxygen, 28% noninvasive ventilation, 5% invasive MV	150 mg aspirin (once daily until discharge) plus SoC *vs*. SoC alone	28 d	Hospitalization length	8 (5–>28) *vs*. 9 (5–>28) d for those discharged alive (5496 *vs*. 5548)
										14 162 (6993 *vs*. 7169)								Severity	1473 *vs*. 1569 MVs/deaths; RR 0.96, 95% CI 0.90–1.03, *P* = 0.23
										14 892 (7351 *vs*. 7541)								Mortality	1222 *vs*. 1299; rate ratio 0.96, 95% CI 0.89–1.04, *P* = 0.35
***Antithrombotics (both anticoagulant and antiplatelet activity)***																			
22	Gonzalez‐Ochoa, AJ^54^	Yes (07‐Mar‐21)	Mexico	Single‐centre double‐blind placebo‐controlled parallel 1:1 RCT	None reported	A site in San Luis Rio Colorado	5 Jun–5 Aug 2020	Adult (>40 years) outpatients with an onset of 3 d or less of suspected COVID‐19 symptoms, with a high calculated risk to develop a severe clinical progression of COVID‐19	No (patients with prolonged anticoagulation in the last 6 mo excluded)	243 (124 *vs*. 119)	NI	55 ± 10 *vs*. 54 ± 11 (for 124 *vs*. 119)	48 *vs*. 46 (for 124 *vs*. 119)	39/18 *vs*. 29/24 (for 124 *vs*. 119)	Outpatients with a high‐level risk to develop severe clinical progression in COVID‐19 according to the COVID‐19 Health Complication calculato (IMSS, Gobierno de Mexico)	Sulodexide (oral 500 lipase releasing units twice daily) or placebo for 21 d	21 d	Hospitalization	22 *vs*. 35 patients needed hospital care; RR 0.60, 95% CI 0.37–0.96, *P* = .03
										57 (22 *vs*. 35) hospitalized								Hospitalization length	6.3 ± 4.1 *vs*. 7.8 ± 4.5 d, *P* = .21
										243 (124 *vs*. 119)								Severity	3 *vs*. 6 invasive MV (RR 0.47, 95% CI 0.12–1.87, *P* = .29); 37 *vs*. 50 patients received supplemental oxygen; RR 0.71, 95% CI 0.50–1.00, *P* = .05
																		Mortality	3 *vs*. 7 deaths; RR 0.41, 95% CI 0.10–1.55, *P* = .19
23	Connors, JM^55^ (ACTIV‐4B)	Yes (11‐Oct‐21)^e^	USA	Multicentre adaptive double‐blind 4‐arm 1:1:1:1 placebo‐controlled RCT	NCT04498273	52 US sites	1 Sep 2020–17 Jun 2021	Symptomatic but clinically stable COVID‐19 outpatients aged 40–80 years.	No	657 (164 *vs*. 165 *vs*. 164 *vs*. 164) aspirin *vs*. prophylactic‐dose apixaban *vs*. therapeutic‐dose apixaban *vs*. placebo	2% Asian, 13% Black, 80% White, 5% other *vs*. 1% Asian, 14% Black, 79% White, 6% other *vs*. 1% Asian, 13% Black, 79% White, 6% other *vs*. 1% Asian, 10% Black, 84% White, 4% other aspirin *vs*. prophylactic‐dose apixaban *vs*. therapeutic‐dose apixaban *vs*. placebo	54 (46–59) *vs*. 55 (46–61) *vs*. 52 (47–58) *vs*. 54 (45–59) aspirin *vs*. prophylactic‐dose apixaban *vs*. therapeutic‐dose apixaban *vs*. placebo	42 *vs*. 42 *vs*. 38 *vs*. 41 aspirin *vs*. prophylactic‐dose apixaban *vs*. therapeutic‐dose apixaban *vs*. placebo	34/18 *vs*. 40/22 *vs*. 35/19 *vs*. 33/15 aspirin *vs*. prophylactic‐dose apixaban *vs*. therapeutic‐dose apixaban *vs*. placebo	All symptomatic but clinically stable COVID‐19 outpatients	Aspirin (81 mg orally once daily), prophylactic‐dose apixaban (2.5 mg orally twice daily), therapeutic‐dose apixaban (5 mg orally twice daily), or placebo for 45 d	45 d	Cardiopulmonary hospitalizations	6 *vs*. 5 *vs*. 5 *vs*. 8 adjudicated hospitalizations (0/144 *vs*. 1/135 *vs*. 2/143 *vs*. 0/136 adjudicated hospitalizations for those who initiated trial therapy)
																		Mortality	0 *vs*. 0 *vs*. 1 *vs*. 1 adjudicated deaths (0/144 *vs*. 0/135 *vs*. 0/143 *vs*. 0/136 adjudicated deaths for those who initiated trial therapy)
***LMDs***																			
24	Davoodi, L^56^	Yes (14‐Sep‐21)	Iran	Single‐centre double‐blind placebo‐controlled parallel 1:1 RCT	IRCT20190727044343N2	Razi referral hospital	26 Jan–17 Feb 2021	Hospitalized (<24 h) COVID‐19 patients aged 20–50 years with CT scan findings for COVID‐19 and respiratory symptoms <10 d	No	40 (20 *vs*. 20)	NI	46 ± 7 *vs*. 46 ± 7	50 *vs*. 55	10/10 *vs*. 20/15	All hospitalized at baseline	Atorvastatin 40 mg *vs*. placebo for 5 d	Until discharge	Hospitalization length	7.95 ± 2.04 *vs*. 9.75 ± 2.29 d, *P* = .012
																		Severity	0 *vs*. 1 MVs
																			3 *vs*. 4 ICU admissions, *P* = .5

^a^ Adults refers to ≥18 years, unless otherwise stated. ^b^ For number of events, the denominator is the reported sample size, unless otherwise indicated. ^c^ Mild defined as blood oxygen saturation of 94% or greater and lung infiltrates ≤50%; moderate, blood oxygen saturation <94%, or lung infiltrates >50%, or ratio of partial pressure of arterial oxygen to fraction of inspired oxygen <300; and severe, invasive mechanical ventilation or haemodynamic instability or multiple organ dysfunction or failure. ^d^ Mild disease includes cases not meeting the criteria for classification as moderate or severe disease; moderate disease was characterised by an oxygen saturation <94%, pulmonary infiltrates >50%, or a partial pressure of oxygen to fractional concentration of oxygen in inspired air ratio <300; and severe disease was defined as respiratory failure, haemodynamic instability, or multiple organ dysfunction. ^e^ Referred to by an expert after the database search (carried out 3 October 2021).

ACEI = angiotensin‐converting enzyme inhibitor; ACEI‐COVID; stopping ACE‐inhibitors in Covid‐19; ACTION = AntiCoagulaTlon cOroNavirus; ACTIV‐4 = A Multicenter Adaptive Randomized Controlled Platform Trial of the Safety and Efficacy of Antithrombotic Strategies in Hospitalized Adults with COVID‐19; ARB = angiotensin receptor blocker; ARDS = acute respiratory distress syndrome; ATTACC = Antithrombotic Therapy to Ameliorate Complications of Covid‐19; BMI = body mass index; BRACE CORONA Blockers of Angiotensin Receptor and Angiotensin‐Converting Enzyme inhibitors suspension in hospitalized patients with coronavirus infection; CCB = calcium channel blocker; CI = confidence intervals; DIC = disseminated intravascular coagulation; DM = diabetes mellitus; ED = emergency department; eGFR = estimated glomerular filtration rate; HEP‐COVID = Efficacy and Safety of Therapeutic‐Dose Heparin *vs*. Standard Prophylactic or Intermediate‐Dose Heparins for Thromboprophylaxis in High‐risk Hospitalized Patients With COVID‐19; HR = hazard ratio; HTN = hypertension; I *vs*. C = interventional *vs*. control; ICU = intensive care unit; INSPIRATION = Intermediate *vs*. Standard‐Dose Prophylactic Anticoagulation in Critically‐ill Patients With COVID‐19: An Open Label Randomized Controlled Trial; IQR = interquartile range; ISTH = International Society on Thrombosis and Haemostasis; LMD = lipid‐modifying drug; MRI = Medical Research Institute; MV = mechanical ventilation; NI = no information; OR = odds ratio; RAPID COVID COAG = Pragmatic Randomised Controlled Trial of Therapeutic Anticoagulation *vs*. Standard Care as a Rapid Response to the COVID‐19 Pandemic; RASTAVI = Renin–Angiotensin System blockade benefits in clinical evolution and ventricular remodeling after Transcatheter Aortic Valve Implantation; RCT = randomized controlled trial; RECOVERY = Randomised Evaluation of COVID‐19 Therapy; REMAP‐CAP = Randomized Embedded Multifactorial Adaptive Platform Trial for Community‐Acquired Pneumonia; REPLACE COVID = Randomized Elimination and Prolongation of ACE inhibitors and ARBs in Coronavirus 2019; RR = risk ratio; SD = standard deviation; SoC = standard of care; SpO_2_ = percent saturation of oxygen; TAVR = transcatheter aortic valve replacement; ULN = upper limit of normal; WHO = World Health Organization.

### 3.2 Qualitative and quantitative synthesis

Table 1 summarises the main results for all 24 studies, while Table 2 provides summary results for the studies included in the quantitative synthesis/meta‐analysis. Table 2 also includes the GRADE strength of evidence rating,^31^ which ranges from moderate to high since all meta‐analyses included at least 1 large properly conducted RCT.

**TABLE 2:** Summary results for studies included in the meta‐analysis

Exposure	Outcome	Meta‐analysis				Only studies with low risk of bias included, Estimate (95%), *I* ^2^	Hypertensive patients Estimate (95%), *I* ^2^
		Included studies (*N)*	Sample size (*n*)	Estimates Estimate (95%), *I* ^2^	Strength of evidence ^a^		
ACEI/ARB (both treatment‐experienced and ‐naïve patients)	Hospitalization length	6	1183	MD ‐0.42 (−1.83; 0.98) d, *I* ^2^ = 51%	High	MD (*N* = 3; *n =* 842): −0.51 (−1.76; 0.75) d, *I* ^2^ = 28%	MD (*N* = 4; *n =* 948): −0.92 (−2.29; 0.45) d, *I* ^2^ = 40%
	Severity	9	1661	RR 0.90 (0.71; 1.15), *I* ^2^ = 24%	High	RR (*N* = 6; *n =* 1320): 0.85 (0.60; 1.20), *I* ^2^ = 38%	RR (*N* = 4; *n =* 948): 0.86 (0.71; 1.04), *I* ^2^ = 0%
	All‐cause mortality	9 (1 with 0 events)	1646	RR 0.92 (0.58; 1.47), *I* ^2^ = 33%	Moderate ^b^	RR (*N* = 5; *n =* 1164): 1.13 (0.70; 1.83), *I* ^2^ = 0%	RR (*N* = 4; *n =* 948): 1.00 (0.60; 1.67), *I* ^2^ = 0%
ARB (recruited patients not taking ACEIs/ARBs)	Hospitalization length	2	111	MD ‐2.32 (−4.81; 0.16) d, *I* ^2^ = 0%	Moderate ^b^	MD (*N* = 1; *n =* 31): −1.00 (−6.13; 4.13) d, *I* ^2^ = NA	MD (*N* = 1; *n =* 80): −2.73 (−5.57; 0.11) d, *I* ^2^ = NA
	Severity	5	589	RR 0.75 (0.42; 1.35), *I* ^2^ = 42%	Moderate ^b^	RR (*N* = 4; *n =* 509): 0.71 (0.30; 1.64), *I* ^2^ = 57%	RR (*N* = 1; *n =* 80): 0.85 (0.36; 1.97), *I* ^2^ = NA
	All‐cause mortality	5 (1 with 0 events)	574	RR 0.53 (0.18; 1.53), *I* ^2^ = 57%	Moderate ^b^	RR (*N* = 3; *n =* 353): 1.23 (0.55; 2.73), *I* ^2^ = 0%	RR (*N* = 1; *n =* 80): 0.38 (0.08; 1.85), *I* ^2^ = NA
ACEI/ARB continuation *vs*. discontinuation	Hospitalization length	4	1072	MD 0.07 (−1.59; 1.73) d, *I* ^2^ = 61%	High	MD (*N* = 2; *n =* 811): −0.38 (−2.08; 1.33) d, *I* ^2^ = 64%	MD (*N* = 3; *n =* 868): −0.55 (−1.97; 0.88) d, *I* ^2^ = 37%
	Severity	4	1072	RR 0.92 (0.76; 1.11), *I* ^2^ = 3%	High	RR (*N* = 2; *n =* 811): 0.87 (0.71; 1.06), *I* ^2^ = 0%	RR (*N* = 3; *n =* 868): 0.86 (0.71; 1.05), *I* ^2^ = 0%
	All‐cause mortality	4	1072	RR 1.23 (0.78; 1.94), *I* ^2^ = 0%	Moderate ^c^	RR (*N* = 2; *n =* 811): 1.08 (0.60; 1.97), *I* ^2^ = 0%	RR (*N* = 3; *n =* 868): 1.12 (0.66; 1.92), *I* ^2^ = 0%
Anticoagulants (DOACS *vs*. placebo)	Hospitalization	2	805	RR 0.82 (0.12; 5.68), *I* ^2^ = 34%	Moderate ^b^	RR (*N* = 1; *n =* 414): 3.43 (0.18; 65.95), *I* ^2^ = NA	NA
Anticoagulants (Therapeutic *vs*. standard/prophylactic)	Hospitalization length	4	1449	MD ‐0.29 (−1.13; 0.56) d, *I* ^2^ = 17%	High	MD (*N* = 4; *n =* 1449): −0.29 (−1.13; 0.56) d, *I* ^2^ = 17%	NA
	Severity	2	2696	RR 0.86 (0.70; 1.04), *I* ^2^ = 0%	High	RR (*N* = 2; *n =* 2696): 0.86 (0.70; 1.04), *I* ^2^ = 0%	NA
	All‐cause mortality	9 (1 with 0 events)	5689	RR 0.93 (0.77; 1.13), *I* ^2^ = 53%	High	RR (*N* = 9; *n =* 5689): 0.93 (0.77; 1.13), *I* ^2^ = 53%	NA

ACEI = angiotensin‐converting enzyme inhibitor; ARB = angiotensin receptor blocker; DOACS = direct oral anticoagulants; *I* ^*2*^ = I‐squared (a heterogeneity measure); MD = mean difference; *N* = number of studies; *n* = sample size; NA = not applicable (<2 studies); RR = risk ratio.

^a^ Based on the GRADE rating.^31^ Evidence from RCTs starts at a high rating and can be downgraded to moderate, low or very low based on risk of bias, imprecision, inconsistency (*I* ^2^ > 70% threshold used), indirectness and publication bias. None of the estimates were downgraded due to including studies with high risk of bias since conclusions remained unchanged when these studies were excluded.

^b^ Estimates downgraded due to imprecision (the effect estimate comes from 1 or 2 small studies, there were few events, or 95% CI include appreciable benefit [RR < 0.75 or harm [RR > 1.25]).^35^

^c^ Estimate became imprecise (95% CI included appreciable benefit/RR < 0.75) when only low risk of bias studies were included.

### 3.3 ACEIs/ARBs

Ten studies^33^, ^34^, ^36^, ^37^, ^38^, ^39^, ^40^, ^41^, ^42^, ^57^ investigated ACEIs/ARBs, with only 1 study (102 patients)^33^ investigating COVID‐19 infectivity. Compared to standard of care, the ACEI ramipril (initial dose 2.5 mg/d, titrated up to 10 mg/d), when newly initiated, was reported to have no effect on COVID‐19 infectivity (hazard ratio 1.15, 95% CI 0.35 to 3.77).

Hospitalization was also investigated by only 1 study (117 patients),^37^ and compared to placebo, newly‐initiated losartan (an ARB, 25 mg once or twice daily, depending on the estimated glomerular filtration rate) given for 10 days, increased the number of hospitalizations (5.2 *vs*. 1.7%, absolute difference of 3.5%) although this was not statistically significant (95% CI −4.8 to 13.2%, *P* = .320).

**FIGURE 2:**
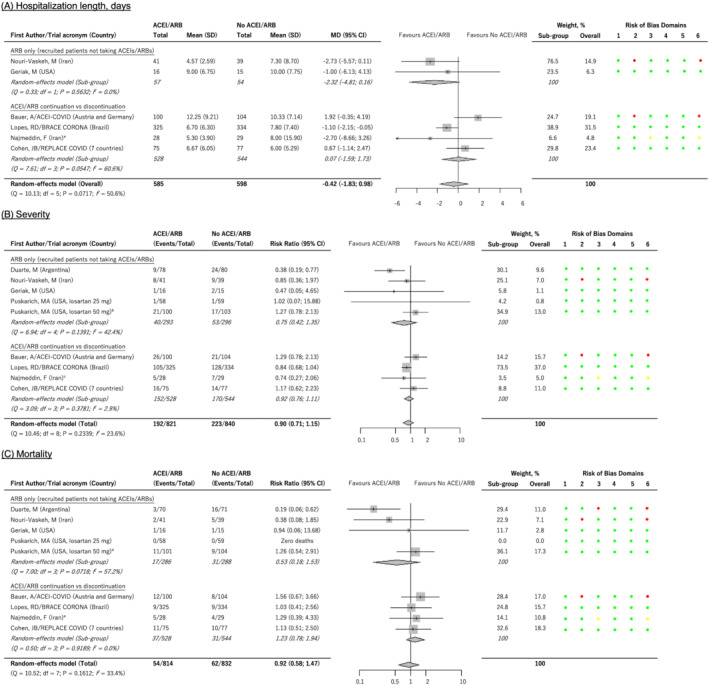
Forest plots for associations between angiotensin‐converting enzyme inhibitors (ACEIs)/angiotensin receptor blockers (ARBs) and COVID‐19 outcomes. ^a^ Total admission days, reported as means (standard deviations), used. When the ‘length of hospital stay’, means (standard deviations) estimated from the reported medians (interquartile range), were used, the subgroup pooled mean difference was 0.41 (95% confidence interval [CI] −1.02 to 1.84, *I* ^*2*^ = 65%) days while the overall pooled mean difference was −0.06 (95% CI −1.36 to 1.24, *I* ^*2*^ = 57%) days. ^b^ Used intubations to define severity as this was more consistent with the rest of the studies. When severity was defined as the requirement for supplemental oxygen, the subgroup and overall pooled risk ratios were 0.74 (95% CI = 0.46 to 1.18, *I* ^*2*^ = 44%) and 0.91 (95% CI 0.78 to 1.07, *I* ^*2*^ = 23%) respectively. ^c^ Used admission to the intensive care unit and/or the requirement for mechanical ventilation to define severity as this was more consistent with the rest of the studies. When severity was defined based on the World Health Organisation COVID‐19 ordinal severity scale, the subgroup and overall pooled risk ratios were 0.93 (0.79 to 1.10, *I* ^*2*^ = 2%) and 0.93 (95% CI 0.75 to 1.14, *I* ^*2*^ = 24%) respectively. ^d^ Follow up of 30 days, which was preferred as this was more consistent with the rest of the studies. When 90‐day follow up was used, the subgroup and overall pooled risk ratios were 0.50 (95% CI 0.20 to 1.29, *I* ^*2*^ = 48%) and 0.89 (95% CI 0.57 to 1.40, *I* ^*2*^ = 31%) respectively. ^e^14‐day follow up. Risk of bias domains: 1 = risk of bias arising from the randomization process; 2 = risk of bias due to deviations from the intended interventions (effect of adhering to intervention); 3 = risk of bias due to missing outcome data; 4 = risk of bias in measurement of the outcome; 5 = risk of bias in selection of the reported result; 6 = overall risk of bias. Colour codes: green = low risk; yellow = some concerns; red = high risk

Seven studies^34^, ^36^, ^38^, ^40^, ^41^, ^42^, ^57^ investigated whether ACEI/ARB exposure (both treatment‐experienced and ‐naïve patients) affected length of hospitalization. One study,^38^ reported only medians and a hazard ratio and could therefore not be included in the primary meta‐analysis. For 6 studies^34^, ^36^, ^40^, ^41^, ^42^, ^57^ (*n* = 1183 patients), ACEIs/ARBs did not influence the duration of hospitalization (mean difference −0.42, 95% CI −1.83; 0.98 d, *I* ^2^ = 51%, Figure 2). When only the 3 estimates^36^, ^40^, ^57^ (842 patients) with low risk of bias (mean difference −0.51, 95% CI −1.76 to 0.75 d, *I* ^2^ = 28%) or the 4 studies^34^, ^40^, ^42^, ^57^ (948 patients) that included hypertensive patients only (mean difference −0.92, 95% CI −2.29 to 0.45 d, *I* ^2^ = 40%) were analysed, the results remained similar (Table 2). Two of the above 6 studies (111 patients) that investigated new initiation of ARBs and compared to amlodipine (a CCB)^34^ or standard of care,^36^ did not show an effect on hospitalization duration (mean difference −2.32, 95% CI −4.81 to 0.16 d, *I* ^2^ = 0%, Figure 2). Conversely, 4^40^, ^41^, ^42^, ^57^ out of the 6 studies (1072 patients) investigated whether discontinuation of ACEI/ARB would influence COVID‐19 outcomes. Again, there was no effect of continuation *vs*. discontinuation of ACEI/ARBs (mean difference in duration of hospitalisation 0.07, 95% CI −1.59 to 1.73 d, *I* ^2^ = 61%). When 2 estimates (811 patients)^41^, ^42^ which did not have a low risk of bias rating were excluded, the pooled effect was similar (mean difference −0.38, 95% CI −2.08 to 1.33 d, *I* ^2^ = 64%). Results did not differ when the 3 studies (868 patients)^40^, ^42^, ^57^ that included only hypertensive patients were analysed (mean difference −0.55, 95% CI −1.97 to 0.88 d, *I* ^2^ = 37%).

The effect of ACEI/ARB exposure (in both treatment‐experienced and ‐naïve patients) on severity (varying definitions based on oxygen supplementation, intensive care unit [ICU] admission, World Health Organization ordinal scale among^43^ others, Table 1) was investigated by 9 studies^34^, ^36^, ^37^, ^38^, ^39^, ^40^, ^41^, ^42^, ^57^ (1661 patients) with ACEIs/ARBs not having an effect on disease severity (pooled RR 0.90, 95% CI 0.71 to 1.15, *I* ^2^ = 24%, Figure 2). When only the 6 estimates^36^, ^37^, ^38^, ^39^, ^40^, ^57^ (1320 patients) with low risk of bias (RR 0.85, 95% CI 0.60 to 1.20, *I* ^2^ = 38%) or 4 studies^34^, ^40^, ^42^, ^57^ (948 patients) that included only hypertensive patients (RR 0.86, 95% CI 0.71 to 1.04, *I* ^2^ = 0%) were pooled, the results were unchanged. Five^34^, ^36^, ^37^, ^38^, ^39^ of the 9 studies (589 patients) investigated ARBs alone in patients not taking ACEIs/ARBs—the analysis showed that, compared to amlodipine,^34^ placebo^37^, ^39^ or standard of care,^36^, ^38^ ARBs had no effect on disease severity (RR 0.75, 95% CI 0.42 to 1.35, *I* ^2^ = 42%, Figure 2), even after 1 estimate^34^ with a high risk of bias was removed (RR 0.71, 95% CI 0.30 to 1.64, *I* ^2^ = 57%, 509 patients). Based on 4 studies^40^, ^41^, ^42^, ^57^ (1072 patients) that investigated ACEI/ARB continuation *vs*. discontinuation, ACEI/ARB continuation did not affect disease severity (RR 0.92, 95% CI 0.76 to 1.11, *I* ^2^ = 3%, Figure 2), even after 2 estimates^40^, ^57^ with a high/some concerns in the risk of bias were excluded (RR 0.87, 95% CI 0.71 to 1.06, *I* ^2^ = 0%, 811 patients). Again, the pooled estimate obtained from only hypertensive patients (3 studies,^40^, ^42^, ^57^ 868 patients) was not different (RR 0.86, 95% CI 0.71 to 1.05, *I* ^2^ = 0%).

Nine trials^34^, ^36^, ^37^, ^38^, ^39^, ^40^, ^41^, ^42^, ^57^ (1646 patients) investigated the association between ACEI/ARB exposure (both treatment‐experienced and ‐naïve patients) and mortality, although 1 study had 0 events.^37^ The results were not statistically significant (pooled RR 0.92, 95% CI 0.58 to 1.47, *I* ^2^ = 33%, Figure 2) even after considering only the 5 estimates^36^, ^37^, ^39^, ^40^, ^57^ with a low risk of bias (RR 1.13, 95% CI 0.70 to 1.83, *I* ^2^ = 0%, 1164 patients). When only hypertensive patients were analysed (4 studies,^34^, ^40^, ^42^, ^57^ 948 patients), the results were similar (RR 1.00, 95% CI 0.60 to 1.67, *I* ^2^ = 0%). Five^34^, ^36^, ^37^, ^38^, ^39^ of the 9 studies (574 patients, 1 study^37^ with 0 events) investigated newly initiated patients on ARBs and showed no effect on mortality risk (RR 0.53, 95% CI 0.18 to 1.53, *I* ^2^ = 57%, Figure 2) even after 2 estimates^34^, ^38^ with a high risk of bias were removed (RR 1.23, 95% CI 0.55 to 2.73, *I* ^2^ = 0%, 353 patients). Four studies (1072 patients)^40^, ^41^, ^42^, ^57^ investigated ACEI/ARB continuation *vs*. discontinuation, and showed that ACEI/ARB continuation had no effect on mortality (RR 1.23, 95% CI 0.78 to 1.94, *I* ^2^ = 0%, Figure 2), even after 2 estimates^40^, ^57^ that did not have a low risk of bias rating were excluded (RR 1.08, 95% CI 0.60 to 1.97, *I* ^2^ = 0%, 811 patients). The pooled estimate obtained from only hypertensive patients (3 studies,^40^, ^42^, ^57^ 868 patients) was similar (RR 1.12, 95% CI 0.66 to 1.92, *I* ^2^ = 0%).

### 3.4 Anticoagulants

Eleven studies^44^, ^45^, ^46^, ^48^, ^49^, ^50^, ^51^, ^52^, ^53^, ^55^ investigated anticoagulant exposure and included large multicentre or multiplatform trials such as the RAPID COVID COAG trial (465 hospitalized patients),^53^ the INSPIRATION trial (562 ICU patients),^44^, ^45^ the ACTION trial (614 hospitalized patients),^47^ the REMAP‐CAP, ACTIV‐4A and ATTACC trials (1098 ICU/critically ill patients^50^ and 2231 hospitalized noncritically ill patients^52^), and the ACTIV‐4B trial (657 outpatients).^48^

**FIGURE 3:**
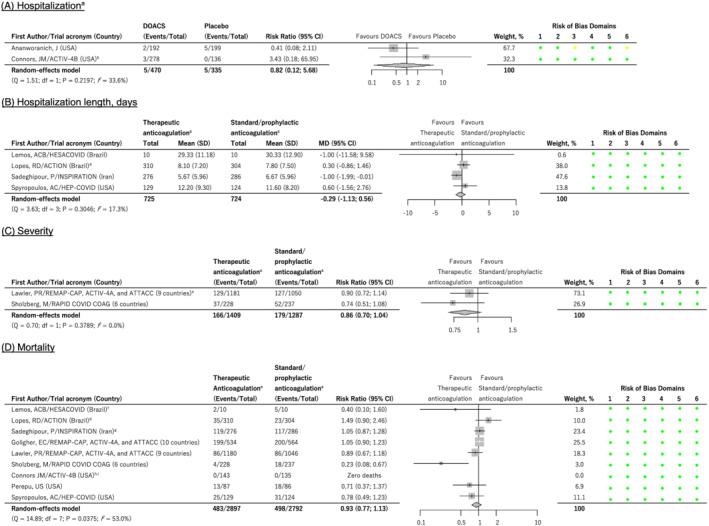
Forest plots for associations between anticoagulants and COVID‐19 outcomes. ^a^ Results are based on those who initiated trial therapy. When the intention‐to‐treat populations are used, the pooled risk ratio becomes 0.55 (95% CI 0.26 to 1.18, *I* ^*2*^ = 0%). ^b^ Active arm is apixaban 2.5 mg or 5 mg orally twice daily. ^c^ Low molecular weight heparin or unfractionated heparin, unless otherwise indicated. ^d^ Therapeutic anticoagulation comprised oral rivaroxaban for stable patients. ^e^ When organ support or death is used as the severity outcome (instead of mechanical ventilation or death), the result becomes significant (risk ratio 0.83, 95% CI 0.72 to 0.95, *I* ^*2*^ = 0%). ^f^ Represents in‐hospital deaths (2 *vs*. 5 deaths). When all‐cause deaths (1 *vs*. 3 deaths) were used instead, the pooled risk ratio (0.94, 95% CI 0.78 to 1.14, *I* ^*2*^ = 51%) remained similar. ^g^ Preferred to Bikdeli *et al*.'s study that used the same dataset (90‐day follow‐up) since Sadeghipour *et al*.'s 30‐day follow‐up was consistent with the rest of the included studies (follow‐up range 21–45 d). Nevertheless, a sensitivity analysis in which the Bidkeli *et al*. study was included instead of the Sadeghipour *et al*. study produced a similar result (pooled risk ratio 0.94, 95% CI 0.77 to 1.14, *I* ^*2*^ = 54%). ^h^ Therapeutic‐dose apixaban (5 mg orally twice daily) *vs*. prophylactic‐dose apixaban (2.5 mg orally twice daily). ^i^ Results are based on those who initiated trial therapy. When the intention‐to‐treat populations are used, the pooled risk ratio becomes 0.94 (95% CI 0.78 to 1.14, *I* ^*2*^ = 48%). DOACS = direct oral anticoagulants. Risk of bias domains: 1 = risk of bias arising from the randomization process; 2 = risk of bias due to deviations from the intended interventions (effect of adhering to intervention); 3 = risk of bias due to missing outcome data; 4 = risk of bias in measurement of the outcome; 5 = risk of bias in selection of the reported result; 6 = overall risk of bias. Colour codes: green = low risk; yellow = some concerns; red = high risk

Two of these trials investigated hospitalization and compared to placebo. Rivaroxaban (10 mg once daily for 21 d)^51^ or apixaban (2.5 or 5 mg twice daily for 45 d)^48^ had no effect on the risk of hospitalization (pooled RR 0.82, 95% CI 0.12 to 5.68, *I* ^2^ = 34%, 805 patients, Figure 3). The ACTIV‐4B trial^48^ also investigated the effect of therapeutic‐dose apixaban (5 mg orally twice daily) *vs*. prophylactic‐dose apixaban (2.5 mg orally twice daily) with the rates of adjudicated cardiopulmonary hospitalizations being similar (1.4 *vs*. 0.7% in those who initiated therapy and 3.1 *vs*. 3.0% in those who were randomized).

Four studies (1449 patients)^44^, ^46^, ^55^, ^57^ investigated the length of hospitalization with exposure to therapeutic, as opposed to prophylactic, anticoagulation doses not altering hospitalization length (mean difference −0.29, 95% CI −1.13 to 0.56 d, *I* ^2^ = 17%, Figure 3). Except for the ACTION trial^47^ that provided the direct oral anticoagulant, rivaroxaban (15 or 20 mg), to clinically stable patients in the therapeutic arm, anticoagulation was by use of low molecular weight heparins (notably enoxaparin) or unfractionated heparin, with the doses determining whether it was therapeutic or prophylactic.

Disease severity was investigated by 3 trials,^51^, ^52^, ^53^ One of these trials^51^ compared rivaroxaban 10 mg once daily for 21 days to a multivitamin supplement provided as the placebo. In this trial and based on the Gates Medical Research Institute scale, 20.7 *vs*. 19.8% (rivaroxaban *vs*. placebo risk difference 1.0, 95% CI −6.4 to 8.4, *P* = .78) and 9.4 *vs*. 11.6% (rivaroxaban *vs*. placebo risk difference −2.2, 95% CI −8.4 to 4.0, *P* = .47) of participants had disease progression in the intention‐to‐treat and modified intention‐to‐treat (only those who took at least 1 dose of the drug and with mild COVID‐19 at day 1) populations, respectively.^51^ The other 2 trials investigated therapeutic *vs*. standard/prophylactic heparin anticoagulation and included a trial that integrated the REMAP‐CAP, ACTIV‐4A and ATTACC platforms,^52^ recruited hospitalized adult noncritically ill/moderate COVID‐19 patients from 9 countries and in which the severity outcome was defined based on the number of invasive mechanical ventilations and/or deaths; and, the RAPID COVID COAG trial,^53^ which recruited hospitalized adult COVID‐19 patients with elevated D‐dimer levels from 6 countries and in which the severity outcome was defined based on ICU admissions, mechanical ventilations and/or deaths. In these 2 studies (2696 patients), therapeutic anticoagulation did not reduce the risk of severe disease (RR 0.86, 95% CI 0.70 to 1.04, *I* ^2^ = 0%, Figure 3) when compared to prophylactic anticoagulation. However, when the severity outcome was defined based on the number of patients who received organ support or died for the REMAP‐CAP, ACTIV‐4A and ATTACC platforms,^52^ the result was statistically significant (RR 0.83, 95% CI 0.72 to 0.95, *I* ^2^ = 0%).

Eleven studies^44^, ^45^, ^46^, ^48^, ^49^, ^50^, ^51^, ^52^, ^53^, ^55^ investigated anticoagulant exposure and mortality, with 2^48^, ^51^ comparing direct oral anticoagulants with placebo. No meta‐analysis was carried out as 1 of the studies that compared rivaroxaban 10 mg against placebo recorded 0 deaths in its 28‐day follow‐up.^51^ In the other study,^48^ the effect of apixaban (2.5 or 5 mg orally twice daily) was similar to placebo (0.0 *vs*. 0.0% deaths in those who initiated therapy and 0.3 *vs*. 0.6% deaths in those who were randomized). Ten studies^44^, ^45^, ^46^, ^48^, ^49^, ^50^, ^52^, ^53^, ^55^ compared therapeutic with prophylactic anticoagulation and 2 of these reported the same population (the INSPIRATION trial), albeit with different follow‐up periods (30^44^ *vs*. 90^45^ d)—with the 30‐day follow‐up being used in the primary analysis as this was consistent with the rest of the included studies (follow‐up range 21–45 d). Therefore, 9 studies (5689 patients), all rated to be of a low risk of bias, were included in the primary meta‐analysis with therapeutic anticoagulation not reducing the risk of death (RR 0.93, 95% CI 0.77 to 1.13, *I* ^2^ = 53%, Figure 3) when compared to standard/prophylactic anticoagulation. In these trials, anticoagulation was achieved using low molecular weight or unfractionated heparins except for the ACTION (used rivaroxaban 15/20 mg in clinically stable patients in the therapeutic arm)^47^ and ACTIV‐4B (used apixaban 5 mg in the therapeutic arm and 2.5 mg in the prophylactic arm)^48^ trials.

### 3.5 Antiplatelet agents

Aspirin, used as an antiplatelet drug, was investigated by 2 studies,^48^, ^54^ including the large sized (*n* = 14 892) platform RECOVERY trial that recruited hospitalized adult COVID‐19 patients from 177 hospitals in the UK, 2 hospitals in Indonesia and 2 hospitals in Nepal.^54^

In the ACTIV‐4B trial,^48^ aspirin 81 mg orally given for 45 days resulted in a similar rate of adjudicated cardiopulmonary hospitalizations when compared to placebo (0.0 *vs*. 0.0% in those who initiated therapy and 3.7 *vs*. 4.9% in those who were randomized) while in the RECOVERY trial,^54^ the median hospitalization length, defined as the time to being discharged alive, was similar in the aspirin and standard care arms (8 *vs*. 9 d, interquartile range for each 5 to >28 d).

The RECOVERY trial,^54^ also investigated severity, with aspirin not having an effect on the number of mechanical ventilations and/or deaths (RR 0.96, 95% CI 0.90 to 1.03, *P* = 0.23).

Both the ACTIV‐4B^48^ and RECOVERY^54^ trials investigated aspirin and mortality outcomes, although we did not conduct a primary meta‐analysis since none of the patients who took at least 1 dose of aspirin/placebo in the ACTIV‐4B trial died within the 45 days of follow‐up. In a secondary analysis that used the ACTIV‐4B intention‐to‐treat population (those randomized to aspirin/placebo), the pooled RR (aspirin *vs*. usual care or placebo) was 0.96 (95% CI 0.90 to 1.04, *I* ^2^ = 0%), with the ACTIV‐4B trial contributing less than 1% of the weight.

### 3.6 Antithrombotics

In addition to anticoagulants and antiplatelets discussed above separately, sulodexide, an antithrombotic with both anticoagulant and antiplatelet activity (given for 21 d) was investigated and it was protective in terms of the risk of hospitalization (RR 0.60, 95% CI 0.37 to 0.96, *P* = .03), although the trial was small with only 243 patients included.^56^ Additionally, it had no effect on hospitalization length (6.3 ± 4.1 d for sulodexide *vs*. 7.8 ± 4.5 d for placebo, *P* = .21), severity/number of invasive mechanical ventilations (RR 0.47, 95% CI 0.12 to 1.87, *P* = .29) and mortality (RR 0.41, 95% CI 0.10 to 1.55, *P* = .19).^56^

### 3.7 CCBs

Amlodipine was investigated as part of the control arm in the Nouri‐Vaskeh *et al*. study^34^ (80 patients, shown under ARBs in Table 1). Compared to losartan, amlodipine was not associated with hospitalization length (7.3 ± 8.7 d for amlodipine *vs*. 4.6 ± 2.6 d for losartan, *P* = .085), severity (9 *vs*. 8 intubations) or mortality (5 *vs*. 2 deaths, *P* = .241).^34^

### 3.8 LMDs

In this drug class, only 1 statin (atorvastatin 40 mg given for 5 d) was investigated, and compared to placebo, decreased hospitalization length (8.0 ± 2.0 *vs*. 9.8 ± 2.3 d, *P* = .012) although it had no effect on severity (3 *vs*. 4 ICU admissions, *P* = .5); however, the trial itself was small sized (*n* = 40 patients).^58^

## 4 DISCUSSION

To update the previously reported associations between cardiovascular drug exposure and COVID‐19 clinical outcomes,^7^ we searched for additional RCTs published between 1 November 2020 and 2 October 2021. Although our previous report included mostly observational studies (427/429, >99%), the conclusions that cardiovascular drugs are not associated with poor COVID‐19 outcomes have been supported by the 24 RCTs that we have included in this update. For example, ACEI/ARB exposure was previously not associated with COVID‐19 severity (odds ratio 1.05, 95% CI 0.81–1.38) or mortality (odds ratio 0.84, 95% CI 0.70–1.00), estimates that lead to conclusions similar to what we report in this update (severity RR 0.90, 95% CI 0.71–1.15; mortality RR 0.92, 95% CI 0.58–1.47). Additionally, subgroup analyses that included only hypertensive patients produced similar estimates, which demonstrates how a, RCT design is able to account for a key confounder that previously remained unadjusted for in the crude estimates we obtained from the observational studies.^7^

Other investigated drugs included anticoagulants, aspirin (an antiplatelet), sulodexide (an antithrombotic with both anticoagulant and antiplatelet activity), amlodipine (a CCB) and atorvastatin (an LMD), although only anticoagulants could be included in the primary meta‐analyses, with therapeutic anticoagulation not affecting hospitalization, hospitalization length, severity and mortality outcomes. Although the number of studies included in the meta‐analyses were small (2, 4, 2 and 9 for hospitalization, hospitalization length, severity and mortality respectively), many of these studies were platform‐based, meaning they could rapidly recruit many patients and the meta‐analyses were therefore well‐sized (805, 1449, 2696 and 5689 for the respective outcomes). Additionally, most of these trials were rated to have a low risk of bias which led to a high strength of evidence for all the outcomes, except hospitalization, which was ranked as moderate. However, and despite this ranking, it is important to emphasize that we did not explore subgroup analyses based on patient characteristics with the exception of the hypertension comorbidity. We also limited our review to the 5 prespecified outcomes, meaning it is possible that therapeutic anticoagulation is beneficial for specific patients based on different clinical endpoints. This was demonstrated by the ATTACC, ACTIV‐4A and REMAP‐CAP Investigators^50^, ^52^ who showed that therapeutic anticoagulation improved the primary outcome of organ support‐free days in noncritically ill COVID‐19 patients, but not in critically ill COVID‐19 patients. Indeed, when we defined the severity outcome based on the number of patients who received organ support or died for the REMAP‐CAP, ACTIV‐4A and ATTACC platforms, therapeutic anticoagulation was protective (RR 0.83, 95% CI 0.72–0.95). However, for the mortality outcome evaluated in this study, the results were not very different among the noncritically ill (RR 0.89, 95% CI 0.67–1.18) and critically ill (RR 1.05, 95% CI 0.90–1.23) patients.

### 4.1 Limitations of this review

The main limitation of this review is the inclusion of relatively few RCTs, despite our comprehensive search strategy, which meant that many drug classes (including β‐blockers, CCBs, diuretics and LMDs) could not be quantitatively synthesized. We were also unable to assess publication bias as we required a minimum of 10 RCTs for each exposure–outcome combination. Nevertheless, this is a recently emerging field and many more RCTs are expected, which will be included in future updates. We did not search trial registries (such as ClinicalTrials.gov or the WHO International Clinical Trials Registry Platform database) for ongoing/expected trials (our literature search in these databases was restricted to trials with results to facilitate timeliness). Nevertheless, 21 RCTs evaluating ACEIs/ARBs were registered as of August 2020,^59^ 75 RCTs evaluating antithrombotic agents were registered as of 16 December 2020,^60^ and 40 RCTs evaluating lipid modulating drugs were registered as of 31 March 2021.^61^ We also relied on single‐reviewer extraction. However, 2 other reviewers verified the accuracy and completeness of the extracted data. In addition to not assessing some efficacy endpoints that are being reported in the literature, we did not assess some safety outcomes (e.g. treatment discontinuation due to bleeding with anticoagulation); however, these were outside the scope of this review. Lastly, and despite the randomization of participants to treatment, residual confounding may exist especially if sample sizes are small^28^ or response‐adaptive randomization is used,^52^ which would require the pooling of adjusted estimates. However, most of the studies included in the meta‐analyses were relatively large and well‐balanced, a reason we rated the strength of evidence as moderate to high.

## 5 CONCLUSIONS

Moderate‐ to high‐certainty RCT evidence suggests that ACEIs/ARBs and therapeutic anticoagulation are not associated with poor COVID‐19 clinical outcomes. However, the routine use of therapeutic anticoagulation is questionable as it offers limited benefits over placebo or standard/prophylactic anticoagulation. There are currently many ongoing RCTs that are expected to be completed/published in the coming months and will be incorporated in our next update, which will be conducted within 6 months of this update. As we wait for more evidence, we suggest that patients with COVID‐19 on cardiovascular drugs should not discontinue taking them as it is very unlikely that these drugs, specifically ACEIs/ARBs, are harmful.

## COMPETING INTERESTS

M.P. has received partnership funding for the following: MRC Clinical Pharmacology Training Scheme (co‐funded by MRC and Roche, UCB, Eli Lilly and Novartis); a PhD studentship jointly funded by EPSRC and Astra Zeneca; and grant funding from Vistagen Therapeutics. He has also unrestricted educational grant support for the UK Pharmacogenetics and Stratified Medicine Network from Bristol‐Myers Squibb and UCB. He has developed an HLA genotyping panel with MC Diagnostics, but does not benefit financially from this. He is part of the IMI Consortium ARDAT (www.ardat.org). None of these of funding sources have been used for the current paper. None of the other authors declared any competing financial interests.

## CONTRIBUTORS

Concept and design: all authors. Acquisition, analysis, or interpretation of data: all authors. Drafting of the manuscript: I.G.A. Critical revision of the manuscript for important intellectual content: S.P., R.M.T., R.K.‐D., A.J. and M.P. Statistical analysis: I.G.A.

## Supporting information


**TABLE S1** Preferred Reporting Items for Systematic Reviews and Meta‐Analyses: The PRISMA Statement.
**TABLE S2** Studies with estimates that were rated as *some concerns* or *high risk* using the revised Cochrane risk‐of‐bias tool.

## Data Availability

All relevant material is provided in main article and supplementary material.
