# Supplementary material for: Cardiovascular drugs and COVID‐19 clinical outcomes: a systematic review and meta‐analysis of randomized controlled trials
Source: Br J Clin Pharmacol. 2022 Apr 25:10.1111/bcp.15331. Online ahead of print. doi: 10.1111/bcp.15331 (PMC9111446; doi:10.1111/bcp.15331)
Supplement: Supplementary file 1 — TABLE S1 Preferred Reporting Items for Systematic Reviews and Meta‐Analyses: The PRISMA Statement. TABLE S2 Studies with estimates that were rated as some concerns or high risk using the revised Cochrane risk‐of‐bias tool. [file BCP-9999-0-s001.docx]

**Cardiovascular drugs and COVID-19 clinical outcomes: a systematic review and meta-analysis of randomized controlled trials**

Innocent G Asiimwe^1^, Sudeep Pushpakom^1^, Richard M. Turner^1^, Ruwanthi Kolamunnage-Dona^2^,

Andrea L. Jorgensen^2^, Munir Pirmohamed^1^.

^1^The Wolfson Centre for Personalised Medicine, MRC Centre for Drug Safety Science, Department of Pharmacology and Therapeutics, Institute of Systems, Molecular and Integrative Biology, University of Liverpool, L69 3GL, Liverpool, United Kingdom. ^2^Department of Health Data Science, Institute of Population Health Sciences, University of Liverpool, L69 3GL, Liverpool, United Kingdom.

Authors for correspondence:

Innocent G. Asiimwe, The Wolfson Centre for Personalised Medicine, Block A Waterhouse Buildings, 1-5 Brownlow Street, Liverpool, L69 3GL. Email: [I.Asiimwe@liverpool.ac.uk](mailto:I.Asiimwe@liverpool.ac.uk)

Professor Sir Munir Pirmohamed, The Wolfson Centre for Personalised Medicine, Block A Waterhouse Buildings, 1-5 Brownlow Street, Liverpool, L69 3GL. Email: [munirp@liverpool.ac.uk](mailto:munirp@liverpool.ac.uk)

**Table of contents**

**Supplementary Tables……………………………………………………………………………….…………………….2**

**Supplementary References……………………………….……………………….…………………………………….5**

**Supplementary Tables**

**Table S1*.* Preferred Reporting Items for Systematic Reviews and Meta-Analyses: The PRISMA Statement^1^**

| **Section and topic** | **Item No** | **Checklist item** | **Section and topic** |
| --- | --- | --- | --- |
| TITLE | | |  |
| Title | 1 | Identify the report as a systematic review, meta-analysis, or both. | Title page |
| ABSTRACT | | |  |
| Structured summary | 2 | Provide a structured summary including, as applicable: background; objectives; data sources; study eligibility criteria, participants, and interventions; study appraisal and synthesis methods; results; limitations; conclusions and implications of key findings; systematic review registration number. | Abstract |
| INTRODUCTION | | |  |
| Rationale | 3 | Describe the rationale for the review in the context of what is already known. | Introduction, paragraphs 1 and 2 |
| Objectives | 4 | Provide an explicit statement of questions being addressed with reference to participants, interventions, comparisons, outcomes, and study design (PICOS). | Introduction, paragraph 2 |
| METHODS | | |  |
| Protocol and registration | 5 | Indicate if a review protocol exists, if and where it can be accessed (e.g., Web address), and, if available, provide registration information including registration number. | Methods, paragraph 1 |
| Eligibility criteria | 6 | Specify study characteristics (e.g., PICOS, length of follow-up) and report characteristics (e.g., years considered, language, publication status) used as criteria for eligibility, giving rationale. | Methods (selection criteria) |
| Information sources | 7 | Describe all information sources (e.g., databases with dates of coverage, contact with study authors to identify additional studies) in the search and date last searched. | Methods (identification of studies) |
| Search | 8 | Present full electronic search strategy for at least one database, including any limits used, such that it could be repeated. | Protocol^2^ |
| Study selection | 9 | State the process for selecting studies (i.e., screening, eligibility, included in systematic review, and, if applicable, included in the meta-analysis). | Methods (data extraction) |
| Data collection process | 10 | Describe method of data extraction from reports (e.g., piloted forms, independently, in duplicate) and any processes for obtaining and confirming data from investigators. | Methods (data extraction) |
| Data items | 11 | List and define all variables for which data were sought (e.g., PICOS, funding sources) and any assumptions and simplifications made. | Methods (data extraction) |
| Risk of bias in individual studies | 12 | Describe methods used for assessing risk of bias of individual studies (including specification of whether this was done at the study or outcome level), and how this information is to be used in any data synthesis. | Methods (assessment of study quality) |
| Summary measures | 13 | State the principal summary measures (e.g., risk ratio, difference in means). | Methods (data synthesis) |
| Synthesis of results | 14 | Describe the methods of handling data and combining results of studies, if done, including measures of consistency (e.g., I^2^) for each meta-analysis. | Methods (data synthesis, heterogeneity measures) |

**Table S1. Continued**

| **Section and topic** | **Item No** | **Checklist item** | **Section and topic** |
| --- | --- | --- | --- |
| Risk of bias across studies | 15 | Specify any assessment of risk of bias that may affect the cumulative evidence (e.g., publication bias, selective reporting within studies). | Methods (publication bias) |
| Additional analyses | 16 | Describe methods of additional analyses (e.g., sensitivity or subgroup analyses, meta-regression), if done, indicating which were pre-specified. | Methods (subgroup analyses) |
| RESULTS | | |  |
| Study selection | 17 | Give numbers of studies screened, assessed for eligibility, and included in the review, with reasons for exclusions at each stage, ideally with a flow diagram. ` | Figure 1 |
| Study characteristics | 18 | For each study, present characteristics for which data were extracted (e.g., study size, PICOS, follow-up period) and provide the citations. | Table 1 |
| Risk of bias within studies | 19 | Present data on risk of bias of each study and, if available, any outcome level assessment (see item 12). | Figures 2-3, Table S2 |
| Results of individual studies | 20 | For all outcomes considered (benefits or harms), present, for each study: (a) simple summary data for each intervention group (b) effect estimates and confidence intervals, ideally with a forest plot. | Table 2, Figures 2-3 |
| Synthesis of results | 21 | Present results of each meta-analysis done, including confidence intervals and measures of consistency. | Table 2, Figures 2-3 |
| Risk of bias across studies | 22 | Present results of any assessment of risk of bias across studies (see Item 15). | None done (<10 studies) |
| Additional analysis | 23 | Give results of additional analyses, if done (e.g., sensitivity or subgroup analyses, meta-regression [see Item 16]). | Tables 2, Figures 2-3 |
| DISCUSSION | | |  |
| Summary of evidence | 24 | Summarize the main findings including the strength of evidence for each main outcome; consider their relevance to key groups (e.g., healthcare providers, users, and policy makers). | Discussion, paragraphs 1-2 |
| Limitations | 25 | Discuss limitations at study and outcome level (e.g., risk of bias), and at review-level (e.g., incomplete retrieval of identified research, reporting bias). | Discussion (limitations of this review) |
| Conclusions | 26 | Provide a general interpretation of the results in the context of other evidence, and implications for future research. | Discussion (conclusions) |
| FUNDING | | |  |
| Funding | 27 | Describe sources of funding for the systematic review and other support (e.g., supply of data); role of funders for the systematic review. | Funding section |

**Table S2*.* Studies with estimates that were rated as ‘some concerns’ or ‘high risk’ using the revised Cochrane risk-of-bias tool.^3^**

| **Study first author** | **Outcome(s)** | **Applicable domains** | **Risk of bias rating** | **Reason(s)** |
| --- | --- | --- | --- | --- |
| Amat Santos, IJ^4^ | Susceptibility to infection | Domain 2 (deviations from the intended interventions (effect of adhering to intervention)) | High risk | Open-label trial, no information on important non-protocol interventions and on the appropriateness of analysis used to estimate the effect of adhering to intervention. |
|  |  | Domain 4 (measurement of the outcome) | High risk | Probably inappropriate outcome method of measurement (many positive patients are asymptomatic so basing on symptoms may miss these patients), patients as outcome assessors (self-reported symptoms) were aware of the treatments they were receiving, and knowledge of the assigned intervention could have influenced the nature and frequency of symptoms reported. |
|  |  | Domain 5 (selection of the reported result) | Some concerns | Outcome was not pre-specified. |
| Nouri-Vaskeh, M^5^ | Hospitalization length, severity, mortality | Domain 2 (deviations from the intended interventions (effect of adhering to intervention)) | High risk | Open-label trial (mentions 'blinding' but patients could tell which drug they were on e.g. different dose schedules), no information on important non-protocol interventions and on the appropriateness of analysis used to estimate the effect of adhering to intervention. |
| Duarte, M^6^ | Mortality | Domain 3 (missing outcome data) | High risk | Data not available for 17 (11%) participants (observed number of events was 19), no evidence that the result was not biased by missing outcome data, and no information that missingness in the outcome depended on its true value. |
| Bauer, A^7^ | Hospitalization length, severity, mortality | Domain 2 (deviations from the intended interventions (effect of adhering to intervention)) | High risk | Open-label trial, no information on important non-protocol interventions co-interventions and on the appropriateness of analysis used to estimate the effect of adhering to intervention. |
| Najmeddin, F^8^ | Hospitalization length, severity (ICU admission, mechanical ventilation), mortality | Domain 3 (missing outcome data) | Some concerns | >10% missing data for hospitalization length, low events except for severity (WHO COVID-19 ordinal endpoint ≥6) and no evidence that the result was not biased by missing outcome data. However, it was not likely that missingness in the outcome depended on its true value. |
| Ananworanich, J^9^ | Hospitalization, hospitalization length, severity, mortality | Domain 3 (missing outcome data) | Some concerns | The observed number of events is not much greater than the number of participants with missing outcome data and no evidence that the result was not biased by missing outcome data. However, it was not likely that missingness in the outcome depended on its true value. |

**Supplementary References**

1. Moher D, Liberati A, Tetzlaff J, Altman DG, Group P. Preferred reporting items for systematic reviews and meta-analyses: the PRISMA statement. *PLoS Med.* 2009;6(7):e1000097.

2. Asiimwe IG, Pushpakom S, Turner RM, Kolamunnage-Dona R, Jorgensen A, Pirmohamed M. Cardiovascular drugs and COVID-19: a living systematic review and meta-analysis (CRD42020191283). *PROSPERO* 2020.

3. Sterne JAC, Savovic J, Page MJ, et al. RoB 2: a revised tool for assessing risk of bias in randomised trials. *BMJ.* 2019;366:l4898.

4. Amat-Santos IJ, Santos-Martinez S, López-Otero D, et al. Ramipril in High Risk Patients with COVID-19. *Journal of the American College of Cardiology.* 2020.

5. Nouri-Vaskeh M, Kalami N, Zand R, et al. Comparison of losartan and amlodipine effects on the outcomes of patient with COVID-19 and primary hypertension: A randomised clinical trial. *International Journal of Clinical Practice.* 2021;75(6).

6. Duarte M, Pelorosso F, Nicolosi LN, et al. Telmisartan for treatment of Covid-19 patients: An open multicenter randomized clinical trial. *EClinicalMedicine.* 2021;37.

7. Bauer A, Schreinlechner M, Sappler N, et al. Discontinuation versus continuation of renin-angiotensin-system inhibitors in COVID-19 (ACEI-COVID): a prospective, parallel group, randomised, controlled, open-label trial. *The Lancet Respiratory medicine.* 2021.

8. Najmeddin F, Solhjoo M, Ashraf H, et al. Effects of Renin-Angiotensin-Aldosterone Inhibitors on Early Outcomes of Hypertensive COVID-19 Patients: A Randomized Triple-Blind Clinical Trial. *American journal of hypertension.* 2021.

9. Ananworanich J, Mogg R, Dunne MW, et al. Randomized study of rivaroxaban vs. placebo on disease progression and symptoms resolution in high-risk adults with mild COVID-19. *Clinical infectious diseases : an official publication of the Infectious Diseases Society of America.* 2021.
